# Putative pseudolysogeny-dependent phage gene implicated in the superinfection resistance of *Cutibacterium acnes*

**DOI:** 10.20517/mrr.2023.42

**Published:** 2024-04-18

**Authors:** Stephanie Wottrich, Stacee Mendonca, Cameron Safarpour, Christine Nguyen, Laura J. Marinelli, Stephen P. Hancock, Robert L. Modlin, Jordan Moberg Parker

**Affiliations:** ^1^Department of Microbiology, Immunology, and Molecular Genetics, University of California Los Angeles, Los Angeles, CA 90024, USA.; ^2^Department of Neurology, Dell Seton Medical Center at the University of Texas at Austin, Austin, TX 78701, USA.; ^3^UCLA Dermatology, University of California Los Angeles, Los Angeles, CA 90095, USA.; ^4^Department of Chemistry, Towson University, Towson, MD 21252, USA.; ^5^Department of Biomedical Science, Kaiser Permanente Bernard J. Tyson School of Medicine, Pasadena, CA 91101, USA.

**Keywords:** *Cutibacterium acnes*, *Propionibacterium acnes*, pseudolysogeny, superinfection resistance, bacteriophage, antibiotic resistance, phage therapy, superinfection exclusion

## Abstract

**Objectives:** *Cutibacterium acnes*, formerly *Propionibacterium acnes*, is a bacterial species characterized by tenacious acne-contributing pathogenic strains. Therefore, bacteriophage therapy has become an attractive treatment route to circumvent issues such as evolved bacterial antibiotic resistance. However, medical and commercial use of phage therapy for *C. acnes* has been elusive, necessitating ongoing exploration of phage characteristics that confer bactericidal capacity.

**Methods:** A novel phage (Aquarius) was isolated and analyzed. Testing included genomic sequencing and annotation, electron microscopy, patch testing, reinfection assays, and qPCR to confirm pseudolysogeny and putative superinfection exclusion (SIE) protein expression.

**Results:** Given a superinfection-resistant phenotype was observed, reinfection assays and patch tests were performed, which confirmed the re-cultured bacteria were resistant to superinfection. Subsequent qPCR indicated pseudolysogeny was a concomitantly present phenomenon. Phage genomic analysis identified the presence of a conserved gene (*gp41*) with a product containing Ltp family-like protein signatures which may contribute to phage-mediated bacterial superinfection resistance (SIR) in a pseudolysogeny-dependent manner. qPCR was performed to analyze and roughly quantify gp41 activity, and mRNA expression was high during infection, implicating a role for the protein during the phage life cycle.

**Conclusions:** This study confirms that *C. acnes* bacteria are capable of harboring phage pseudolysogens and suggests that this phenomenon plays a role in bacterial SIR. This mechanism may be conferred by the expression of phage proteins while the phage persists within the host in the pseudolysogenic state. This parameter must be considered in future endeavors for efficacious application of *C. acnes* phage-based therapeutics.

## INTRODUCTION

*Cutibacterium acnes*, formerly *Propionibacterium acnes*, is a gram-positive bacterium of the human epidermal microbiome. It has been documented widely within human preclinical lesions, also called microcomedones, regardless of skin microflora variability^[1-3]^. Certain strains of *C. acnes* have been implicated as key contributors to acne vulgaris (i.e., acne)^[2,3]^. Though generally considered a mild affliction, many individuals affected by acne may suffer from psychosocial problems and physical pain at affected sites^[2]^. Additionally, *C. acnes* may contribute to severe health complications such as post-operative prosthetic hardware contamination, sarcoidosis, spondylodiscitis, prostate pathologies, and even Parkinson’s Disease^[4,5]^. Extensive time and effort have been dedicated to the study of *C. acnes* and *C. acnes* bacteriophages, the viruses that infect *C. acnes*, to better understand and characterize the predominant strains in humans, particularly those correlated with pathogenicity^[1,3]^. Some studies such as those investigating biofilm dynamics have evaluated the efficacy of phage therapy against *C. acnes* and have demonstrated bacterial resistance to infection in certain cases^[6-9]^. The leading proposal for this phenomenon in *C. acnes* was that clustered regularly interspaced short palindromic repeat (CRISPR) elements conferred resistance in a Cas protein-dependent manner^[3,10]^. Since then, experimental results aiming to induce CRISPR-mediated resistance in clinical strains of *C. acnes* have suggested inconsistency with the notion of CRISPR as an exclusive immunity mechanism, indicating other mechanisms are also at play^[10,11]^.

An alternative cause of bacteriophage (phage) resistance is the mechanism of superinfection exclusion (SIE). SIE is a property conferred to the host bacterium via expression of certain temperate phage proteins which give rise to superinfection resistance (SIR)^[12-14]^. SIE conferring SIR has been documented among both viruses and phages such as those that infect *Pseudomonas aeruginosa*, *Escherichia coli*, and *Salmonella typhimurium*^[15-20]^. Several other phages that infect *Streptococcus thermophilus* and *Lactococcus lactis* including TP-J34 and TP-778L have also been found to display SIE conferring SIR, and proteins involved in the mechanism have been identified and explored^[13,20-23]^. The SIR phenotype has been observed in *C. acnes* by a failure of new plaque formation when phages that were previously able to cause lysis were re-spotted onto bacterial lawns composed of bacteria that had proliferated within prior plaque centers^[11]^. The ability of phages to persist within a host cell supports the possibility of an SIE mechanism dependent on the expression of *SIE* gene(s) from a latent phage genome. While *C. acnes* phages do not undergo a classic lysogenic state as they do not encode integrases, they are known to exhibit pseudolysogeny in a state either characterized by the phage genome remaining as an episome, or in a state of inefficient lysis^[6,11,23-25]^. Despite documentation of *C. acnes* phage capacity to both undergo pseudolysogeny and to confer SIR, an SIE mechanism has not been characterized and the mechanism conferring SIR has not been described.

In the present study, *C. acnes* phage Aquarius was isolated from the facial microcomedones of a donor without a history of acne. An early host range assay revealed the SIR phenotype in which bacterial proliferation was observed in the centers of Aquarius plaque clearings on bacterial lawns of *C. acnes* ATCC 6919. This observation prompted a search for SIE characteristics, including verifying Aquarius’s ability to undergo and maintain pseudolysogeny^[11,23]^. Sequencing and genomic annotation of the Aquarius genome was performed, and bioinformatics were used to identify and attempt characterization of a putative *SIE* gene. Evidence suggesting a phage-mediated SIE phenotype could begin to shed light on shortcomings of past phage therapy experiments, as well as present a target for future studies aimed at fine-tuning phage therapies for the purpose of controlling pathological *C. acnes* presence.

## METHODS

### *C. acnes* host strains and bacteriophages

*C. acnes* ATCC 6919, clinical isolates [strains 060PA1, 110PA3, and 020PA1, described in Fitz-Gibbon *et al.* (2013)], and cultures of putative pseudolysogens were cultivated as described previously by Marinelli *et al.* by incubation at 37 °C for three days under anaerobic conditions using the AnaeroPack System (Mitsubishi Gas Chemical Company, Tokyo, Japan)^[1,3]^. For all subsequent experiments, *C. acnes* plates were incubated under these conditions, unless otherwise noted. *C. acnes* bacteriophage isolates were isolated and purified from facial microcomedones using Bioré® pore strips (Kao USA Incorporated, Cincinnati, OH, USA) applied to the nose as previously described^[1,3]^. The microcomedone samples were scraped off of the strip and re-suspended in 1 mL of liquid A Media agar (12 g casitone, 12 g yeast extract, 4 g D+ glucose, 4 g KH_2_PO_4_, 1 g magnesium sulfate heptahydrate, ddH_2_O up to 1.0 L). The inoculated A Media was then passed through a 0.22-micron filter to isolate all particles smaller than 0.22 microns, including phages. The filtered sterilized contents were then added to 500 μL *C. acnes* culture and plated on A Media hard agar via the soft agar overlay technique using 0.5% A Media Top Agar (liquid A Media + 5 g agar). A control of *C. acnes* and SM buffer (10 mL 1M Tris stock at pH 7.5, 10 mL MgSO_4_ stock, 4 g NaCl, 970 mL ddH_2_O, 10 mL of CaCl_2_ stock) was also plated. After obtaining plaques, samples were taken from eight plaques and plated with *C. acnes* on A Media hard agar. The phage lysate used throughout the study was obtained by flooding the web lysis plate from this isolation (using the sample that initially generated the most plaques) with reinforced clostridial media (RCM) buffer and then collecting and filtering the lysate. A plaque assay was performed to determine the titer of the lysate, and a dilution scheme was generated to yield complete lysis, web lysis, and countable plaque plates using ten-fold serial dilutions of the phage at 10^-1^, 10^-2^, 10^-3^, 10^-4^, and 10^-5^. This process was conducted twice using the highest titer dilution web lysis plate to generate a high-titer phage lysate. Bacteriophages used in this study are described in Table 1^[3,23,26-28]^.

**Table 1:** *C. acnes* bacteriophages used in this study

**Phage name**	**GenBank Accession No.**	**Bacterial host**	**Experiments**
Aquarius^a^	MF919491	*Cutibacterium acnes*	All experiments
Lauchelly^a^	NC_027628	*Cutibacterium acnes*	Cross spotting; bioinformatics
BruceLethal^a^	NC_031084	*Cutibacterium acnes*	Cross spotting
QueenBey^a^	NC_031005	*Cutibacterium acnes*	Cross spotting
ATCC 29399B_C^b^	JX262225	*Cutibacterium acnes*	Cross spotting; escape mutant isolation
P100A^c^	JX262221	*Cutibacterium acnes*	Cross spotting
P100D^c^	NC_018852	*Cutibacterium acnes*	Cross spotting
P104A^c^	NC_018845	*Cutibacterium acnes*	Cross spotting
P105^c^	NC_018849	*Cutibacterium acnes*	Cross spotting
TP-J34^d^	HE861935	*Streptococcus thermophilus*; *Lactococcus lactis*	Bioinformatics
TP-778L^e^	HG380752	*Streptococcus thermophilus*; *Lactococcus lactis*	Bioinformatics

a Source: UCLA Advanced Research in Virology Undergraduate Laboratory Curriculum^[26]^; ^b^Source: Clear/lytic (C) plaque isolated by Marinelli *et al.* (2012) from a mixed population of clear and turbid plaques observed from *C. acnes* phage stock ATCC 29399B originally described by Webster and Cummins (1978)^[3,27]^; ^c^Source: Marinelli *et al.* (2012)^[3]^; ^d^Source: Neve *et al.* (2003)^[28]^; ^e^Source: Ali *et al.* (2014)^[23]^. UCLA: University of California, Los Angeles.

### Electron microscopy

The phage sample was prepared by placing an aliquot of phage lysate on a carbon EM-grid and staining with 1% uranyl acetate (0.1 g uranyl acetate, 10 mL ddH_2_O). Images were taken with a Philips CM120 electron microscope (F.E.I. Company, Hillsboro, OR, USA).

### Viral DNA purification and sequencing

Phage DNA was isolated by incubating a sample of phage lysate with 5 mg/mL DNase I and 10 mg/mL RNase A for 30 min. The sample was then treated with Promega Wizard® DNA Clean-Up System (Madison, Wisconsin) as described by the manufacturer’s protocol to isolate viral DNA. The concentration of the isolated phage DNA was obtained using a NanoVue spectrophotometer (GE Healthcare, Chicago, Illinois). The phage genomes were sequenced using an Illumina MiSeq (Illumina, San Diego, CA, USA) and assembled using the software program Newbler (Roche, Branford, Connecticut, USA) at the Pittsburgh Bacteriophage Institute in Pennsylvania as previously described^[29]^.

### Genome annotation and comparative analysis

Preliminary annotation of the genome was conducted via the prokaryotic gene protein-coding potential prediction software tools Glimmer and GeneMark, in conjunction with DNA Master as the point source for genomic edits and organization^[30-32]^. Refining of the locations of the auto-called genes was performed using a set of bioinformatics tools, including Starterator, Phamerator, and the NCBI BLAST suite^[33,34]^. Following confirmation of all gene locations, functional assignments were performed for each gene using a variety of bioinformatics tools, including the domain predicting tool HHPred, the Conserved Domain Database (CDD), Phamerator, the NCBI BLAST suite, Phagesdb (local) BLAST, and the Protein Database (PDB)^[34-38]^.

Gene Content Similarity (GCS) for the *C. acnes* phages used in this study was calculated using the Explore Gene Content tool embedded in the Acinobacteriophage Database (https://phagesdb.org/genecontent/). GCS is calculated by identifying the number of phams (gene “phamilies” with a high degree of alignment) that are present in both phages and dividing that number by the total number of phams present in each phage, then averaging the two values^[34]^. Phamerator.org was used to generate comparative genomic maps for the *C. acnes* phages^[34,38]^. The *streptococcus* phages were not included in these analyses because the Phagesdb and Phamerator databases are limited to actinobacteriophages. Pairwise comparisons of the genome nucleotide sequences for all phages in Table 1, including the *streptococcus* phages, were conducted using the Genome-BLAST Distance Phylogeny (GBDP) method^[39]^ under settings recommended for prokaryotic viruses^[40]^. The resulting intergenomic distances were used to infer a balanced minimum evolution tree with branch support via FASTME including SPR postprocessing^[41]^ for the D0 formula. Branch support was inferred from 100 pseudo-bootstrap replicates each. Trees were rooted at the midpoint^[42]^ and visualized with iTOL^[43]^.

### SIR testing

Lawns of *C. acnes* ATCC 6919 and three clinical isolates [strains 060PA1, 110PA3, and 020PA1, described by Fitz-Gibbon *et al.* (2013)] were spot inoculated with phage lysates and observed for bacterial regrowth within the plaques^[1]^. Putative pseudolysogens were collected by taking five samples of bacteria that grew in the centers of areas of clearing, three from a host range assay and two from a phage lysate plate. These samples were inoculated in RCM and incubated for three days at 37 °C under anaerobic conditions. The putative pseudolysogens were plated on A Media and 10-fold dilutions (ranging from 10^-1^ to 10^-9^) of phage lysates were spotted on the lawns to test for SIR.

### Lysogen patch testing

Isolated *C. acnes* bacteria displaying SIR phenotypes were assayed for the pseudolysogeny phenotype. *C. acnes* ATCC 6919 was plated on A Media hard agar via the soft agar overlay technique and the putative pseudolysogens were then streaked onto these plates and monitored for spontaneous phage release after incubation. Negative control plates were prepared for each putative pseudolysogen by streaking the bacterial samples on plates without ATCC 6919 to ensure the growth of the streaked bacteria. For the stability patch tests, the same techniques were employed while serially streaking the individual strains onto a lawn of ATCC 6919 bacteria every three days over the course of approximately six months (approximately 60 passages).

### Pseudolysogeny PCR

Pseudolysogeny PCR, as described by Liu *et al.* (2015), was performed to identify if the isolated lysogens harbored the Aquarius genome^[11]^. A master mix was prepared using the forward primer 5’-CCG AAG CCG ACC ACA TCA CAC C-3’ and the reverse primer 5’-TCA TCC AAC ACC TGC TGC TGC C-3’. DNA from uninfected bacteria and DNA-free negative controls were also assayed. All amplicons were run on a 0.8%-0.9% agarose gel for 25 min at 100 V. A Fisher’s exact test was conducted to assess independence between PCR results and patch test phenotype.

### Aquarius gp41 protein bioinformatics

BLASTp was conducted against the Phagesdb.org database using the sequence of *ltp* from phages TP-J34 and TP-778L^[22,23,33]^. The EMBL-EBI protein sequence and classification tool InterPro was then used to analyze the signature profiles of Ltp from phages TP-778L and TP-J34, as well as gp41 of phages Lauchelly (Genbank Accession number KR337650) and Aquarius^[44]^. Multiple Em for Motif Elicitation (MEME) was employed to search for putative motifs in the non-cytoplasmic domains identified by InterPro and BLASTp^[45]^. The protein alignment and phylogeny analysis tool Mega7 was used to identify residues with conserved charge within the putative active site domains of the *C. acnes* Ltp-like proteins by conducting a protein alignment of LtpTP-778L and LtpTP-J34 with several *C. acnes* phage gp41 proteins^[46]^. The web portal for protein structure and function prediction RaptorX was used to compare overall predicted disorder between gp41 and Ltp^[47]^. Aquarius gp41 structure prediction was performed with AlphaFold2^[48]^ and structural homology was predicted with DALI^[49]^. Surface electrostatic potentials were calculated with the Adaptive Poisson-Boltzmann Solver^[50]^, structural alignment was performed with TM-Align^[51]^, and protein structure images were generated using PyMOL (Schrödinger, LLC).

### Escape mutant isolation

Isolation of escape mutants was attempted by incubating 10 μL of phage Aquarius lysate or phage ATCC 29399B_C (Genbank Accession JX262225) lysate with bacterial lysogen strains for 30 min, followed by plating for lawns using the soft agar overlay technique. Lysate dilutions of 10^-0^ and 10^-1^ were used as experimental groups, with dilution spots of 10^-2^, 10^-4^, 10^-6^, and 10^-7^ on a lawn of ATCC 6919 as controls.

### *C. acnes* phage infection and qPCR

To analyze RNA levels at various stages in the infection cycle, bacterial cultures of *C. acnes* ATCC 6919 and an Aquarius pseudolysogen were grown in RCM and diluted to an OD_600_ of 0.2 (approximately 1 × 10^8^ CFU/mL). Phage-free ATCC 6919, the pseudolysogen, and ATCC 6919 plus Aquarius at a multiplicity of infection of 10 were incubated at 37 °C for the 90-minute duration of the active infection period. Total RNA was isolated using a Qiagen RNeasy® Kit (QIAGEN Group, Valencia, CA, USA) and analyzed for purity via Bioanalyzer. Following the isolation, cDNA was generated from the RNA according to the Bio-Rad iSCRIPT cDNA kit protocol using a Bio-Rad thermocycler (Bio-Rad Laboratories, Inc.; Hercules, CA, USA). After obtaining cDNA, qPCR was performed with primers for Aquarius gp41 and ATCC 6919 RecA reference gene [Table 2] on undiluted cDNA using the Roche KAPA SYBR FAST qPCR kit (Roche, Basel, Switzerland). A one-way ANOVA with post hoc Tukey HSD was used to assess statistical significance.

**Table 2:** qPCR primers for expression level analysis

	**qPCR primers**
ATCC 6919 RecA forward	5’-GAC CGT TAA GAT CGC CGC TA-3’
ATCC 6919 RecA reverse	5’-CGT GCT CGG CGT CAA TAA AG-3’
Aquarius gp41 forward	5’-CTC CCT ACA AGC CGA ACA GG-3’
Aquarius gp41 reverse	5’-AGG TGT CTT TGT GAG CTC CG-3’

## RESULTS

### Phage Aquarius characterization

**Figure 1:**
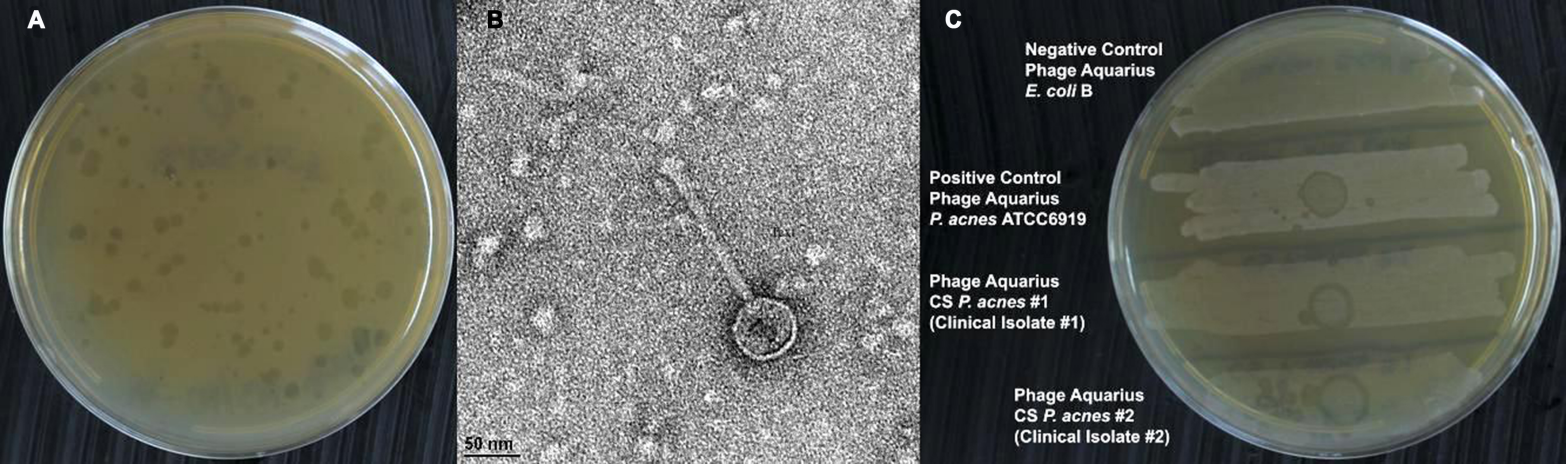
Phage Aquarius Characterization. (A) Plaque morphology of Aquarius was of variable size with clear to turbid plaques. The plate image was enhanced by 30% in brightness and contrast to show detail; (B) Transmission electron microscopy at 52000X magnification. The length of the phage tail and the diameter of the phage head were measured using the software program ImageJ^[52]^ and were found to be 150.3 and 58.8 nm, respectively. The presence of the long non-contractile tail and the icosahedral head are characteristic of Caudoviricetes phages; (C) SIR phenotype characterized by the growth of bacteria in the center of an area of clearing following spot inoculation of phage Aquarius on cultures of *C. acnes* ATCC 6919 (second row streak) and two *C. acnes* clinical isolates (third and fourth row streaks) during an initial host range assay. No bacterial lysis was observed for the negative control spot inoculation on *E. coli* (first row streak). SIR: superinfection resistance.

Aquarius, which was isolated from the microcomedones of a donor individual, displayed variable plaque sizes with clear to turbid morphologies on *C. acnes* ATCC 6919 [Figure 1A]. Aquarius virions were visualized using transmission electron microscopy and displayed a morphology similar to other previously isolated *C. acnes* phages [Figure 1B]^[52]^. After initial isolation of the phage, an early host range experiment additionally demonstrated the SIR phenotype plaque morphology as has been described previously by Liu *et al.* (2011) [Figure 1C]^[11]^.

**Figure 2:**
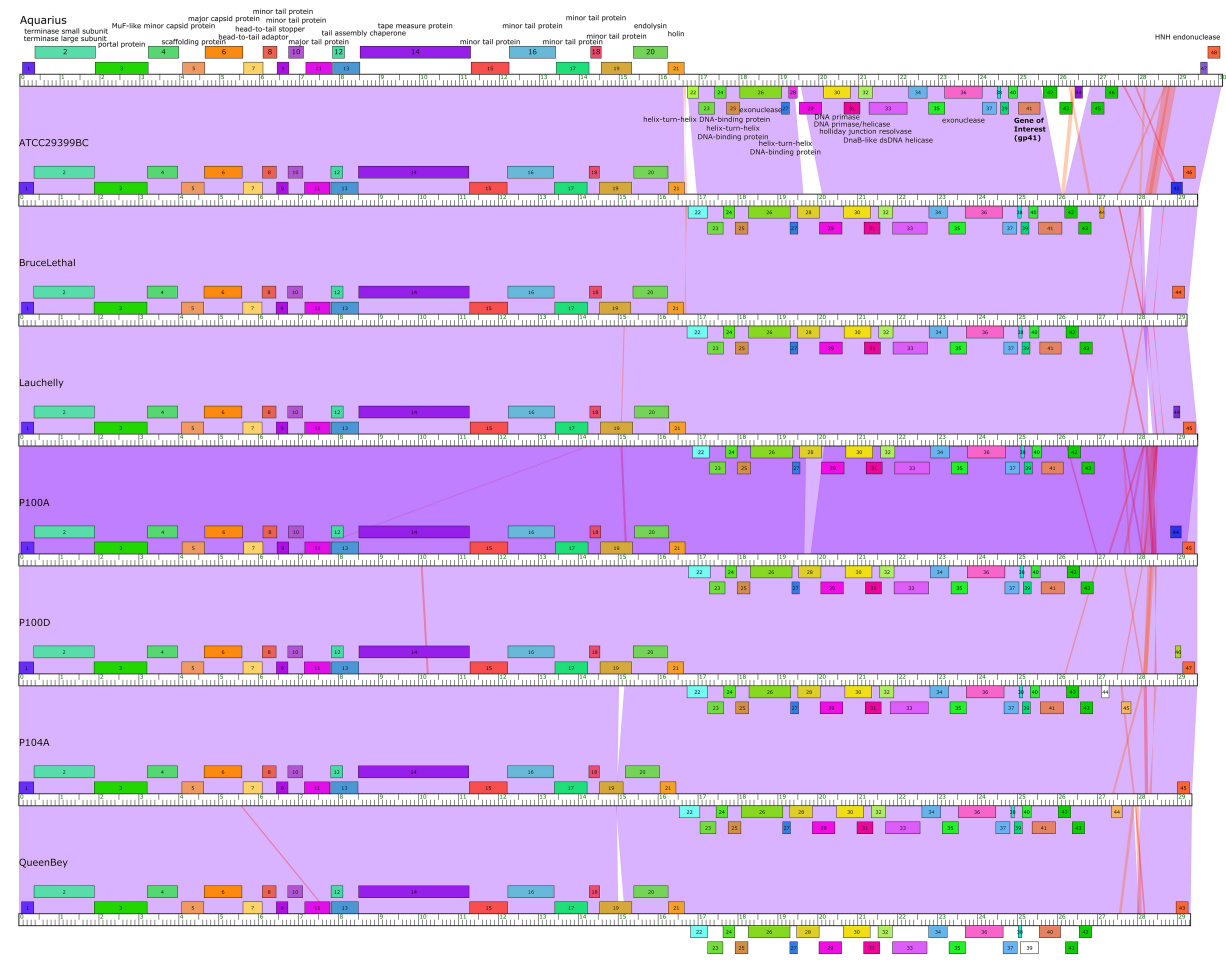
Phamerator Comparative Genomics Maps. Comparative genomic maps generated using Phamerator.org for the eight *C. acnes* phages used in this study. Each genome is arranged along a kilobase ruler with forward transcribed genes marked above the ruler and reverse transcribed genes marked below the ruler. Each gene product is color coded by related protein “phamilies” or “phams” determined by BLASTP and ClustalW as described by Cresawn *et al.* (2011)^[34]^. Phams with known functions are labeled along the Aquarius genome map. Nucleotide sequence similarity based on BLASTN is shown by the shaded regions between genomes, and is colored based on its E value, with violet representing the best matches (lowest E values) and red the worst matches (highest E values). White areas indicate that there is no nucleotide similarity in those regions. As reported for previously studied *C. acnes* phages, the phages used in this study have genomes with a high degree of synteny and nucleotide conservation, as demonstrated by the mostly violet shading between genomes^[3,6,11]^.

**Figure 3:**
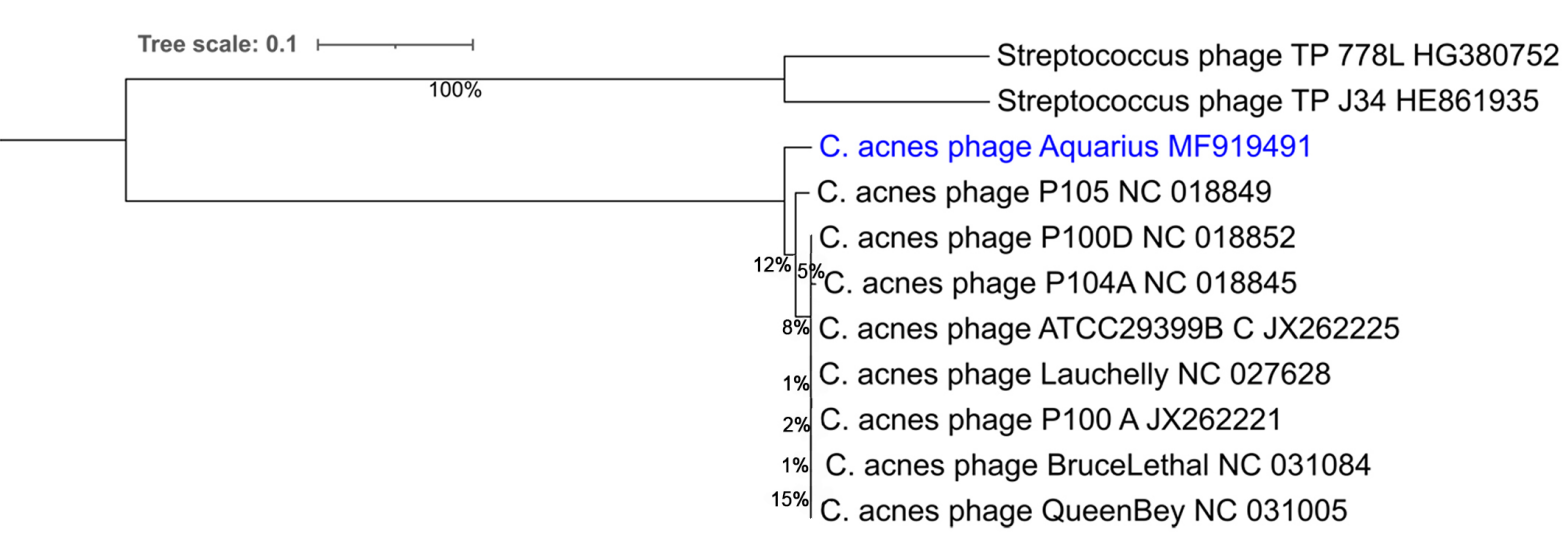
Genome-BLAST Distance Phylogeny tree for *C. acnes* and *S. thermophilus* phages. The numbers above the branches are GBDP pseudo-bootstrap support values from 100 replications. The branch lengths of the resulting trees are scaled in terms of the recommended VICTOR formula (D0)^[40]^. GBDP: Genome-BLAST Distance Phylogeny.

Genomic analysis demonstrated a genome of 30112 bp with 54.5% GC content and 48 putative ORFs were identified. The genome ends have 11 base 3’ sticky overhangs (TCGTACGGCTT), suggesting that the genome is capable of the circularization necessary for pseudolysogeny^[3,11,24]^. Comparative genomics of the representative *C. acnes* phage genomes used in this study [Table 1] indicated 86%-96.7% GCS with a high degree of synteny and nucleotide conservation [Table 3 and Figure 2]^[34]^. These genome characteristics were demonstrative of homology with other previously isolated *C. acnes* phage genomes, which are known to have very limited diversity^[11]^. This was further supported by the GBDP that were calculated for all of the phages in Table 1, which included Aquarius and eight representative *C. acnes* phages, along with two *Streptococcus thermophilus* phages to root the tree [Figure 3]. All the *C. acnes* phages exhibited a high degree of genomic homology, as evidenced by the short branch lengths and low bootstrap values.

**Table 3:** *C. acnes* phage gene content similarity

**Phage**	**ATCC29399BC**	**Lauchelly**	**Bruce lethal**	**Queen bey**	**P100A**	**P100D**	**P104A**	**P105**
Aquarius	87.3	88.3	89.3	86	88.3	86.3	88.3	90.4
ATCC29399BC		94.5	95.6	92.2	96.7	92.5	94.5	92.3
Lauchelly			96.6	93.2	95.6	93.5	95.6	93.3
BruceLethal				94.3	96.6	94.6	96.6	94.4
QueenBey					93.2	91.3	93.2	91
P100A						93.5	95.6	93.3
P100D							95.7	91.4
P104A								93.3

### Evidence for pseudolysogeny and stability

The identification of 3’ sticky overhangs suggested the genome’s capacity to undergo circularization and exist as a pseudolysogen. To further assess for pseudolysogenic characteristics, putative Aquarius pseudolysogens were streaked (patched) onto an uninfected bacterial lawn. Areas of clearing were observed surrounding the bacterial patches, indicating spontaneous phage release following lytic induction of pseudolysogenized phage. PCR primers that anneal to the end of *C. acnes* phage genomes were used to assess if circularized phage genomes were present in the bacterial samples [Supplementary Figure 1]. Gel electrophoresis showed characteristic bands at roughly 735 base pairs (corresponding to the size of the overlapping portion of the phage genome that was amplified) for all putative pseudolysogens. No bands were produced on the uninfected ATCC 6919. These findings were consistent with previous experiments demonstrating the capacity of *C. acnes* phages to undergo pseudolysogeny^[11,24]^.

**Figure 4:**
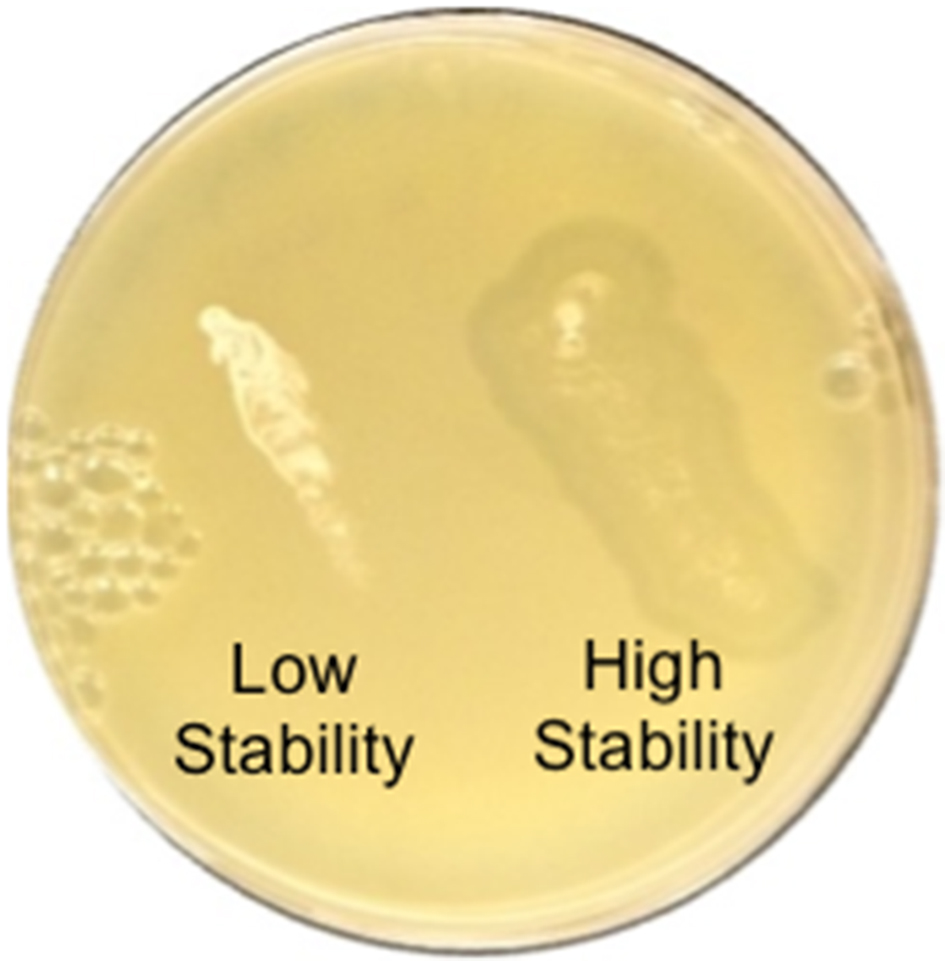
High Stability/Low Stability Patch Test Samples, derived from passage 4, on ATCC 6919. The above picture illustrates the capacity of the passaged lysogenic bacteria taken from the fourth pass of the patch test to produce an area of clearing surrounding the lysogen streak (high stability group, on the right). Also shown is a prior putative pseudolysogen that possessed the ability to cause lysis but lost its lysing capacity after the fourth passage (low stability group, on the left). Positive and negative controls were also prepared (lawn of pure ATCC 6919, and putative pseudolysogens with no bacterial lawn, respectively), and demonstrated successful bacterial growth (not shown).

Pseudolysogens are generally known to be less stable than full lysogens and, therefore, at greater susceptibility of being diluted out over time^[24]^. Given this, it was considered that the numerous lytic centers scattered on the pseudolysogen lawns may be a manifestation of induction of the lytic life cycle in bacteria after sequential passaging. After initial patch testing, sub-isolates of two pseudolysogens with either a persistently sustained (“high”) or quickly lost (“low”) capacity for spontaneous lytic phage release were assessed for pseudolysogen stability via serial patch testing for phage release over the course of several months [Figure 4]. The sub-isolates of the “low stability” group lost the ability to lyse the surrounding lawn after six total passages, which corresponded to about 20 days. This isolate was the first to lose its lysing capacity after beginning serial patch testing, as demonstrated by the lack of an area of clearing surrounding the bacterial patch on the bacterial lawn. The “high stability” group, however, sustained lytic capacity for over six months of passages, corresponding to roughly 180 days. It was at this point that the study was stopped.

### Pseudolysogeny PCR

Pseudolysogeny PCR was performed on both the high- and low-stability groups as described above to assay for maintenance of the circularized genome using the primers described by Liu *et al.*^[11]^. All 27 high stability group sub-isolates produced a 735 bp band indicative of the presence of the phage genome, while the two representative samples for the low stability group did not, indicating true loss of the phage genome. A Fisher’s exact test was conducted to assess independence between PCR result and patch test phenotype, and the results indicated a strong association between them (*P* < 0.002) [Table 4]. Additionally, viral spot testing on cultures of the high- and low-stability groups after reaching five months of passages revealed lysis of the low-stability group, but complete SIR in the high-stability group, supporting the hypothesis of a pseudolysogeny-associated phage resistance mechanism.

**Table 4:** Bacterial SIR correlates with phage pseudolysogeny, and susceptibility to infection

**Test**	**Outcome**	**Uninfected (control)**	**Infected with sustained SIR**	**Infected with loss of SIR**
PCR	735 bp band produced	0	27	0
	No 735 bp band produced	1	0	2
Spot test	Susceptible to infection	1	0	1
	Not susceptible to infection	0	1	0

The SI-resistant phenotype was observed in bacteria that yielded a 735 bp band on PCR, indicating that the bacteria with this phenotype contain a phage undergoing pseudolysogeny. The bacteria no longer exhibiting this phenotype were not associated with a band produced on PCR, nor were they resistant to infection when challenged with Aquarius. SIR: superinfection resistance.

### Evidence for SIR

When phage Aquarius was spotted onto lawns of *C. acnes* ATCC 6919, bacterial proliferation was observed in the center of areas of clearing. To test for SIR, bacteria were isolated from plaque centers, cultured, and used to create bacterial lawns for spot tests with ten-fold dilutions of the Aquarius phage lysate. The spot tests revealed no evidence of lysis on the lawn of previously infected bacteria, indicating SIR. Additionally, putative Aquarius lysogens of three *C. acnes* clinical isolates demonstrated SIR by phage Aquarius [Table 1]. To assess the range of cross-immunity that the SIR mechanism may confer, eight additional representative *C. acnes* phages that were previously isolated and characterized were spot tested for their ability to infect putative Aquarius lysogens, and no lysis was observed on the lawns for any of the *C. acnes* phages tested [Table 1]. To further test the range of this SIR mechanism, putative ATCC 6919 lysogens for seven different *C. acnes* phages were isolated and cross-tested for SIR for a variety of phages [Table 1]. No lysis was observed for any of the tested combinations of pseudolysogens and re-infecting phages, suggesting a mechanism for broadly preventing superinfection by *C. acnes* phages.

### Identification of Ltp-like protein

In addition to genome annotation, bioinformatics was used to characterize gene(s) of unknown function that are putatively involved in phage-mediated SIR. Previous research has shown that the Ltp protein in *Streptococcus thermophilus* and *Lactococcus lactis* phages TP-J34 and TP-778L confers SIR via an electrostatic interaction between Ltp and the tape measure protein C-terminus, resulting in a stalling of the ejection complex and prevention of infection^[22,23]^. The BLASTp for Ltp of TP-J34 and TP-778L yielded a hit with a protein of unknown function (gp41) in *C. acnes* phage Lauchelly, as well as in *Propionibacterium freudenreichii* phages PFR1 and PFR2 [Table 5]^[33]^.

**Table 5:** BLASTp results for Ltp-like proteins

**Phage**	**Host**	**Ltp_TP-J34_score**	**Ltp_TP-J34_E-value**	**Ltp_TP-J34_identities (%)**	**Ltp_TP-778L_score**	**Ltp_TP-778L_E-value**	**Ltp_TP-778L_identities (%)**
Lauchelly	*C. acnes*	30	1.1	26/124 (20)	31	0.83	26/121 (21)
PFR1	*P. freudenreichii*	110	6e-25	65/138 (47)	108	4e-24	66/149 (44)
PFR2	*P. freudenreichii*	110	6e-25	63/138 (47)	108	4e-24	66/149 (44)

**Figure 5:**
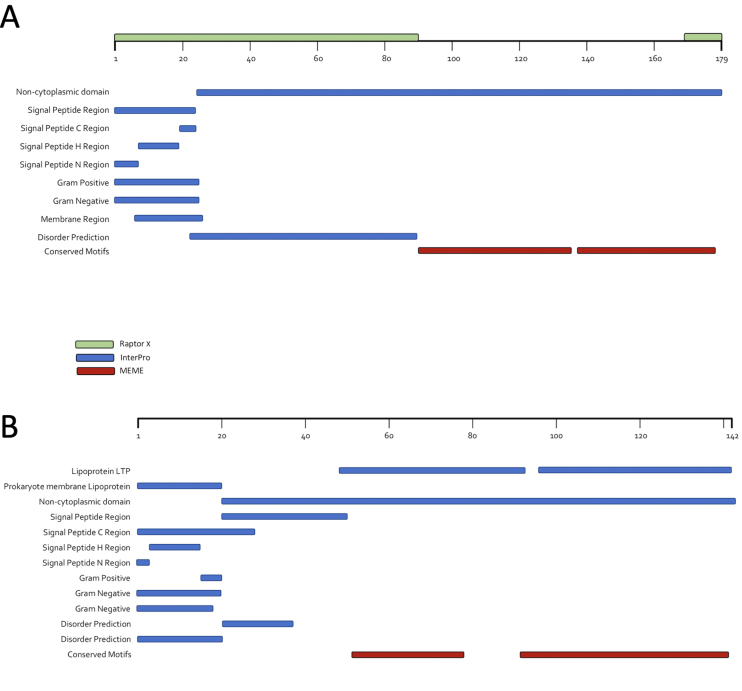
Protein signatures of Phage TP-J34 Ltp and Phage Aquarius gp41. (A) Phage TP-J34 Ltp. Output from InterPro (blue) indicated the presence of several protein signatures, including the two conserved domains that comprise the active site region of Ltp (residues 49-92 and 96-141). Other notable signatures included a prokaryotic lipoprotein (residues 1-20), regions of disorder (residues 21-50 and 21-37), signal peptide H-region (residues 4-15), signal peptide C-region (residues 16-20), signal peptide N-region (residues 1-3), signal peptide (residues 1-20), transmembrane signal peptide (residues 1-28), and a non-cytoplasmic domain (residues 21-142). Output from MEME (red) indicated the presence of two conserved motifs as well, spanning residues 46-77 and 91-141. Output from RaptorX (green; depicted on ruler) also identified a generally high region of disorder spanning from the first residue to roughly residue 50, and a small region at the very end of the peptide spanning roughly one to two residues; (B) Protein signatures of Phage Aquarius gp41. Output from InterPro (blue) indicated the presence of several notable protein signatures, including regions of disorder (residues 23-89 and 34-51), signal peptide H-region (residues 8-19), signal peptide C-region (residues 20-24), signal peptide N-region (residues 1-7), signal peptide (residues 1-24), transmembrane signal peptide (residues 1-25), transmembrane helix (residues 7-26), and a non-cytoplasmic domain (residues 25-179). Output from MEME (red) indicated the presence of two conserved motifs as well, spanning residues 84-133 and 137-177. Output from RaptorX (green; depicted on ruler) also identified a generally high region of disorder spanning from the first residue to roughly residue 91, and a small region at the very end of the peptide spanning roughly three to five residues. MEME: Multiple Em for Motif Elicitation.

InterPro was used to compare signatures between LtpTP-J34, LtpTP-778L, and gp41 of Lauchelly and Aquarius^[44]^. There was a remarkably similar signature profile between gp41 and Ltp, most noticeably the conservation of a long non-cytoplasmic domain at a similar locus and relative length within the sequences, as well as several signal peptide signatures. A roughly 90 amino acid-long region of disorder was also predicted in Ltp and gp41 by InterPro and RaptorX, corresponding to the region after the end of the signal peptide at its C-terminal region and the beginning of the region within the non-cytoplasmic domain with which the first HTH domain of Ltp begins^[44,47]^. MEME was employed to search for putative motifs within these non-cytoplasmic domains, considering a hallmark of Ltp family proteins is the presence of two repeat HTH domains. The output for TP-J34 Ltp and TP-778L Ltp compared to gp41 from sixteen *C. acnes* phages demonstrated two motifs of similar size with low *P*-values overlapping the non-cytoplasmic domain^[22,45]^. Lastly, Mega7 was used to identify charge conserved residues within the putative active site domains by conducting a protein alignment of TP-778L and TP-J34 Ltp proteins with several *C. acnes* phage gp41 proteins^[46]^. The output indicated the presence of several charge conserved residues that may fit with the model of the Ltp-Tape Measure Protein (TMP) interaction according to Bebeacua *et al.* (2013), although further study is required to make definitive claims about active roles for any specific residue(s)^[22]^. The combined findings of InterPro, MEME, and RaptorX are visually depicted in Figure 5, which correspond to the outputs for Ltp of TP-J34 and gp41 of Aquarius, respectively.

**Figure 6:**
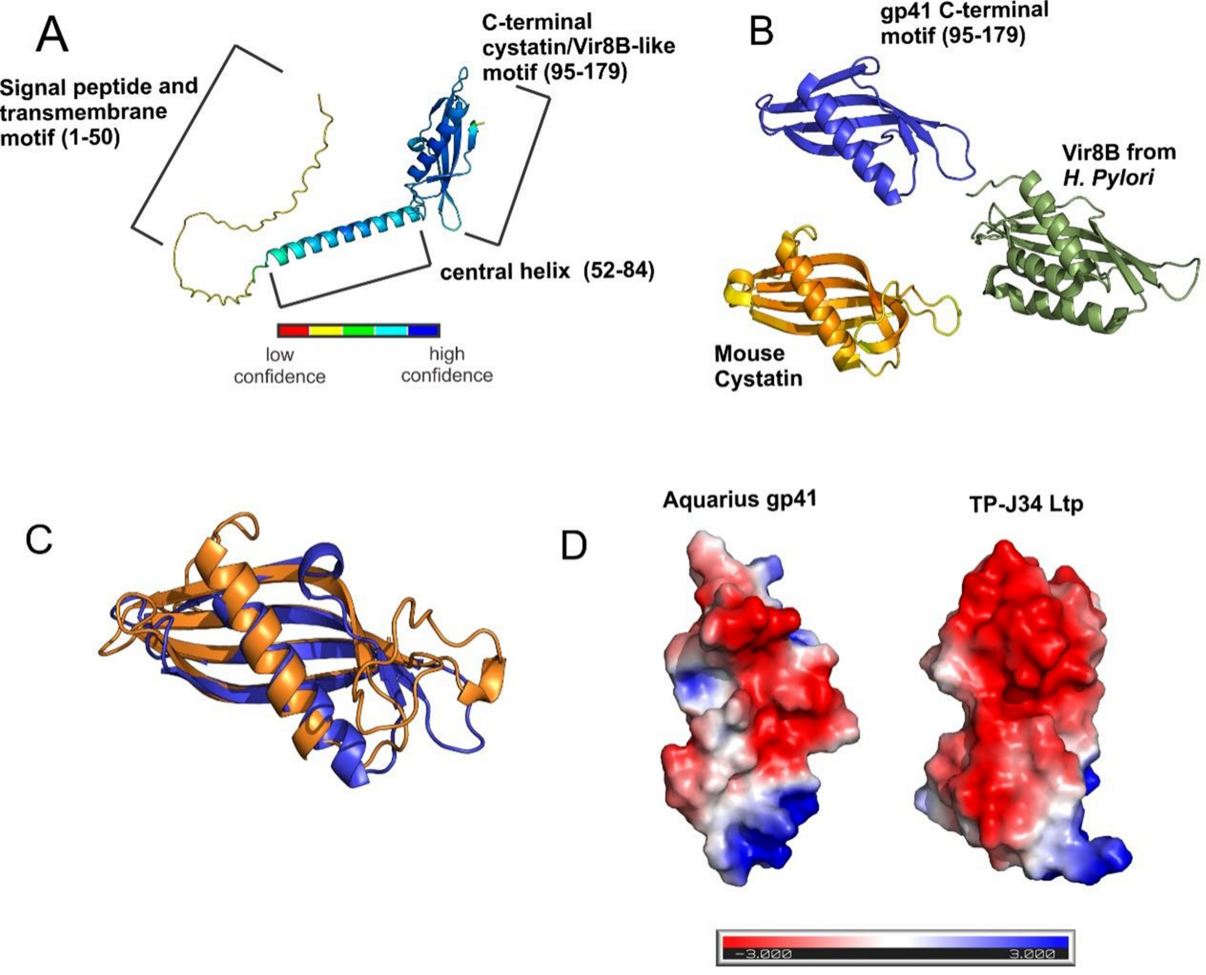
The predicted structure of Aquarius gp41 resembles factors that facilitate protein-protein interactions. (A) Aquarius gp41structure predicted by AlphaFold2. Structure is colored by confidence and functional motifs are labeled; (B) Closest structural homologs to Aquarius gp41 as determined by the DALI structural homology server - Vir8B (Green: pdbid is 6IQT) and mouse Cystatin (orange:pdbid 6UIO); (C) Structural alignment between gp41 95-179 (blue) and mouse cystatin (orange); (D) Electrostic potential was solved using the APBS for Aquarius gp41 (left) and TP-J34 Ltp (right). Electrostatic potential scale is given in kT/e where negative (red) and positive (blue) surface potentials are shown. APBS: Adaptive Poisson-Boltzmann Solver.

Protein structure prediction with AlphaFold2 (AF2) indicates that the N-terminal residues of Aquarius gp41 (1-50) do not adopt a high-confidence structure. This correlates with the disorder prediction by InterPro and RaptorX [Figure 5B and Figure 6A]. The gp41 N-terminal region is also predicted to contain a signal peptide and transmembrane motif that targets gp41 to the cell membrane [Figure 5B and Figure 6A]. Gp41 residues 52-84 adopt a confidently predicted α-helical element that connects the N-terminal transmembrane sequence to a confidently predicted C-terminal element that is predominantly β-conformation (95-179) but includes a single α-helix (94-112) [Figure 5A and Figure 6B]. Secondary structure predicted by AF2 agrees with PsiPRED and Spider predictions implemented through the Max Plank Institute (MPI), Quick2D server (not shown). A structural homology search using DALI shows that the Aquarius gp41 C-terminal motif (95-179) adopts a structure similar to extracellular matrix proteins cystatin/latexin and VirB8-like proteins of type IV secretion systems with Z scores of 8.9 and 7.0 indicating a high likelihood of structural similarity, respectively - gp41 aligns with mouse cystatin with a RMSD of 2.14 Å [Figure 6C]. Notably, cystatins have been shown to assemble into nonpathological amyloid matrices that are thought to be involved in cell maturation and protection^[53,54]^. Additionally, Vir8B is an essential structural component of type IV secretion systems with a periplasmic motif (res 86-226) involved in the assembly of these multiprotein complexes^[55]^. It is possible that gp41 facilitates SIE by assembling a protective matrix around the pseudolysogen, protecting them from infection or by directly interacting with and inhibiting the activity of proteins required for secondary phage infection. Gp41 surface electrostatics were calculated with the Adaptive Poisson-Boltzmann Solver (APBS) which shows a patch of negatively charged surface analogous to that observed for the TP-J34 Ltp [Figure 6D]. Thus, it may be that gp41 is involved in SIE through interactions with phage TMP, similar to the proposed mechanism described for *S. thermophilus* phage TP-J34 Ltp.

### No escape mutants found

Since bioinformatics suggested the presence of a conserved Ltp-like protein in Aquarius’s genome, three attempts were made to isolate escape mutant phages capable of infecting pseudolysogens. It was hypothesized that after several reinfection attempts, pseudolysogens conferring SIR may eventually acquire SIR-compromising mutations mapping to either gp41 or the tape measure protein of Aquarius, which would support an SIE mechanism characteristic of that described by Bebeacua *et al.* (2013)^[22]^. However, all attempts at plating Aquarius on pseudolysogens were unsuccessful in producing plaques, indicating a tight immunity mechanism at play.

### qPCR of Aquarius Ltp-like gp41

**Figure 7:**
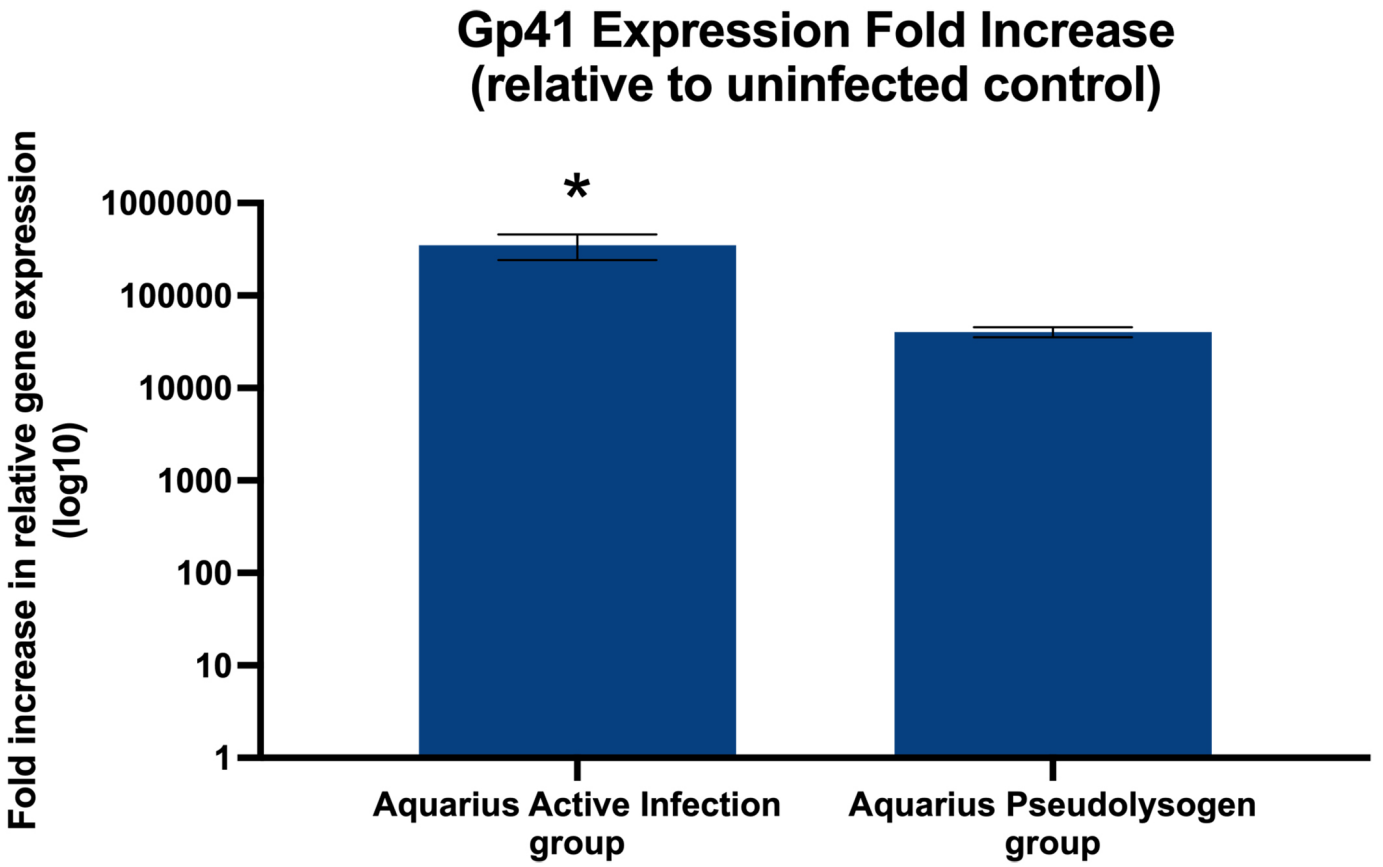
Gp41 expression relative to the uninfected control. The average fold increase in phage Aquarius gp41 mRNA expression averaged over three PCR trials, normalized to the bacterial housekeeping gene *RecA*. A one-way ANOVA with post hoc Tukey HSD comparison indicated a significant difference between the active infection and pseudolysogen groups (^*^P < 0.05). Error bars the Standard Error.

To find evidence linking gp41 to SIR, qPCR was performed to assess gene expression in a phage-free control compared to actively infected bacteria and Aquarius pseudolysogens. The gp41 expression was normalized to the bacterial housekeeping gene *RecA* [Table 2]. The average results of three qPCR trials indicated a fold increase of approximately 333,000 times more expression of Aquarius gp41 in the active infection group and 40,000 times more expression in the pseudolysogen group compared to the phage-free control [Figure 7]. This represents an expression ratio of 8.3:1 for the active phage infection to the pseudolysogen. A one-way ANOVA with post hoc Tukey HSD comparison of the qPCR results yielded a significant result (*P* < 0.05), suggesting an important role for gp41 in early phage infection. Thus, it may be that gp41 is highly expressed during initial infection, the period in which many individual phages have recently entered their bacterial hosts and are undergoing replication, whereas expression may be maintained at a lower level during the pseudolysogenic life cycle.

## DISCUSSION

The results of this research support the previous findings that Aquarius and other *C. acnes* phages can undergo pseudolysogeny and support the hypothesis of a SIR mechanism conferred via expression of an Lpt-like gene (*gp41*) from a semi-stable phage episome within the bacterial host. Gene mapping has demonstrated that *C. acnes* phages have little genomic diversity, which may account for the effectiveness of the SIR mechanism against other *C. acnes* phages in cross spot testing^[3,11]^. Bioinformatics demonstrated the presence of gp41, a conserved protein in all *C. acnes* phage genomes tested, which bears structural resemblance to the known phage SIR protein Ltp. Gp41 was also expressed at high levels during active infection, as well as in established pseudolysogen strains as demonstrated by qPCR. The lower gp41 expression in latent pseudolysogen samples relative to the sharp increase in the newly infected samples may suggest the breaching of a saturation point or feedback mechanism that results in waning gp41 expression over time. However, without further data, definitive conclusions cannot be drawn about the precise mechanism, nor the evolutionary implications of the SIE phenomenon in *C. acnes* phages as described here. A future study assessing the expression of gp41 in other *C. acnes* phages, as well as the expression of Ltp in TP-J34 and/or TP-778L, may provide further support for a similar mechanism if a similar expression pattern were to be found. Additionally, there are various proposals as to the evolutionary etiology of SIR in general which may also be applicable with regard to the pattern of waning expression over time throughout the phage life cycle. Recent studies have demonstrated that in some cases of SIE, superinfecting phages are still able to initiate the lytic life cycle but produce fewer progeny phages per time (lower multiplicity of infection). Thus, rather than being completely inhibited from superinfecting, these secondary infecting phages have lower fitness than the initial phage that was infected^[56,57]^. Other phages, such as T4 which infects *E. coli*, have SIE properties as well, although the primary benefit of this phenomenon is lengthening the phage latent period, which produces a larger burst size^[49]^. Perdoncini Carvalho *et al.* (2022) describe a “bottleneck, isolate, amplify, select” (BIAS) mechanism which characterizes SIE as a beneficial trait by blocking all but a few viral genome copies from undergoing intracellular replication^[58]^. This allows for beneficial mutations to propagate by blocking highly homologous but less fit phage genomes from replicating, in addition to promoting a larger burst size as previously described^[56-58]^. Studies involving mapping of induced gp41 mutations to reduced efficacy of SIE would support this phenomenon, as well as aid with identifying key residues involved in SIE if gp41 is in fact a key driver. These phage mechanisms contrast with the direct phage cleavage CRISPR-Cas system that is documented among several strains of *C. acnes* and promotes resistance to infection. Utilization of this system, however, presents an option for the creation of genomes with induced gp41 mutations, as well as the production of gene knockouts^[59,60]^.

Despite these findings, it remains unconfirmed whether the mechanism of gp41 in Aquarius is governed by a mechanism paralleling that of Ltp, which is characterized by negatively charged residues on the Ltp surface interacting with predominantly positively charged surface peptides on TMP during phage DNA ejection into its host^[22]^. It is intriguing that the gp41 non-cytoplasmic C-terminal does contain a negatively charged surface analogous to that present in Ltp, indicating the potential for functional similarities [Figure 6D]. It is notable that other phages, such as T5 which infects *E. coli*, also have SIE proteins that have been characterized, which promote SIE by other mechanisms such as by inducing a conformational change upon the formation of an outer membrane receptor protein complex that blocks host receptors allowing for phage internalization in superinfection^[18]^. However, it may be postulated that given Aquarius is a gram-positive bacteriophage, its structural resemblance to SIE proteins in phages infecting gram-negative bacteria may be limited. A detailed study aimed at eliciting the crystal structure of gp41 is warranted for direct comparison with the protein structures of other SIE proteins (including Ltp). However, it is notable that membrane glycoproteins are notoriously difficult to crystallize using traditional X-ray crystallography owing to their non-soluble properties. This has been a limiting factor in other studies aimed at determining the crystal structure of other glycoproteins, although vapor diffusion crystallography is an alternative technique that has reported greater success^[61]^. Should gp41 be involved in an SIE mechanism analogous to that of Ltp, it may be difficult to isolate.

Ideally, characterization of this protein would be confirmed via studies in which recombineered phage strains that do not possess gp41 (i.e., protein knockouts) are isolated as described above, and *C. acnes* strains are infected with them to observe for the SIE phenotype, including strains of *C. acnes* preeminently engineered to contain gp41. However, cloning via electroporation in *C. acnes* bacteria is currently not possible^[62]^. Alternatively, escape mutant studies may be reperformed on a larger scale, potentially with concomitant use of a mutagen such as what was done in the study conducted by Leavitt *et al.* (2023), to try to induce mutations that localize to gp41 as an alternative to inducing targeted mutants, e.g., with the use of CRISPR-Cas systems^[19,59,60]^. Should escape mutant studies in the future or recombineering prove successful in isolating phage with a mutated gp41 or gp41 knockout, x-ray crystallography of potential target proteins (including the tape measure protein) would be warranted to solidify understanding of the gene’s mechanism in *C. acnes* phages on a bio-molecular level. We do find that predictive artificial intelligence modeling such as with Alphafold^[48]^ provides some insights into the prospective function of gp41 in SIR, specifically, that gp41 structure resembles factors that facilitate protein-protein interactions to assemble large extracellular matrices or macromolecular complexes. This could indicate a role for gp41 in SIR through modulation of aspects of phage adsorption or DNA release. A lack of similarity to known phage SIR systems might suggest that the mechanism for Aquarius-mediated systems is uncharacterized and novel.

Our work, along with previous research, demonstrates that there are likely multiple evolved mechanisms governing *C. acnes* resistance to phage infection, including the CRISPR-Cas system, restriction-modification systems, and SIE mechanisms^[11,59,60,62,63]^. An understanding of the mechanism(s) by which this resistance may be produced in bacteria that were previously capable of being lysed by phage may give rise to the exploitation of the phenomenon in the fine-tuning of phage-based therapeutics. Likewise, this approach would give rise to a more thorough understanding of infection in the context of the evolutionary life cycles that the phage can undergo to promote its propagation and the success of its survival.

## DECLARATIONS

### Availability of data and materials

The full annotated genome of phage Aquarius may be found at https://phagesdb.org/phages/Aquarius/.

### Copyright

© The Author(s) 2024.

## References

[1] Fitz-Gibbon S, Tomida S, Chiu BH (2013). *Propionibacterium acnes* strain populations in the human skin microbiome associated with acne. J Invest Dermatol.

[2] Li H

[3] Marinelli LJ, Fitz-Gibbon S, Hayes C (2012). *Propionibacterium acnes* bacteriophages display limited genetic diversity and broad killing activity against bacterial skin isolates. mBio.

[4] Perry A, Lambert P (2011). Propionibacterium acnes: infection beyond the skin. Expert Rev Anti Infect Ther.

[5] Leheste JR, Ruvolo KE, Chrostowski JE (2017). *P. acnes*-driven disease pathology: current knowledge and future directions. Front Cell Infect Microbiol.

[6] Brüggemann H, Lood R (2013). Bacteriophages infecting *Propionibacterium acnes*. Biomed Res Int.

[7] Jończyk-Matysiak E, Weber-Dąbrowska B, Żaczek M (2017). Prospects of phage application in the treatment of acne caused by *Propionibacterium acnes*. Front Microbiol.

[8] Coenye T, Peeters E, Nelis HJ (2007). Biofilm formation by *Propionibacterium acnes* is associated with increased resistance to antimicrobial agents and increased production of putative virulence factors. Res Microbiol.

[9] Holmberg A, Lood R, Mörgelin M (2009). Biofilm formation by *Propionibacterium acnes* is a characteristic of invasive isolates. Clin Microbiol Infect.

[10] Brüggemann H, Lomholt HB, Kilian M (2012). The flexible gene pool of *Propionibacterium acnes*. Mob Genet Elements.

[11] Liu J, Yan R, Zhong Q (2015). The diversity and host interactions of *Propionibacterium acnes* bacteriophages on human skin. ISME J.

[12] Labrie SJ, Samson JE, Moineau S (2010). Bacteriophage resistance mechanisms. Nature Rev Microbiol.

[13] Sun X, Göhler A, Heller KJ, Neve H (2006). The *ltp* gene of temperate *Streptococcus thermophilus* phage TP-J34 confers superinfection exclusion to *Streptococcus thermophilus* and *Lactococcus lactis*. Virology.

[14] Seed KD (2015). Battling phages: how bacteria defend against viral attack. PLoS Pathog.

[15] McAllister WT, Barrett CL

[16] Hofer B, Ruge M, Dreiseikelmann B (1995). The superinfection exclusion gene (sieA) of bacteriophage P22: identification and overexpression of the gene and localization of the gene product. J Bacteriol.

[17] Mahony J, McGrath S, Fitzgerald GF, van Sinderen D (2008). Identification and characterization of lactococcal-prophage-carried superinfection exclusion genes. Appl Environ Microbiol.

[18] (2022). van den Berg B, Silale A, Baslé A, Brandner AF, Mader SL, Khalid S. Structural basis for host recognition and superinfection exclusion by bacteriophage T5. Proc Natl Acad Sci U S A.

[19] Leavitt JC, Woodbury BM, Gilcrease EB, Bridges CM, Teschke CM, Casjens SR (2024). Bacteriophage P22 SieA mediated superinfection exclusion. mBio.

[20] Hasan M, Ahn J (2022). Evolutionary dynamics between phages and bacteria as a possible approach for designing effective phage therapies against antibiotic-resistant bacteria. Antibiotics.

[21] Ruiz-Cruz S, Parlindungan E, Erazo Garzon A (2020). Lysogenization of a lactococcal host with three distinct temperate phages provides homologous and heterologous phage resistance. Microorganisms.

[22] Bebeacua C, Lorenzo Fajardo JC, Blangy S (2013). X-ray structure of a superinfection exclusion lipoprotein from phage TP-J34 and identification of the tape measure protein as its target. Mol Microbiol.

[23] Ali Y, Koberg S, Heßner S (2014). Temperate Streptococcus thermophilus phages expressing superinfection exclusion proteins of the Ltp type. Front Microbiol.

[24] Lood R, Collin M (2011). Characterization and genome sequencing of two *Propionibacterium acnes* phages displaying pseudolysogeny. BMC Genomics.

[25] Cieślik M, Bagińska N, Jończyk-Matysiak E, Węgrzyn A, Węgrzyn G, Górski A (2021). Temperate bacteriophages - the powerful indirect modulators of eukaryotic cells and immune functions. Viruses.

[26] Shapiro C, Moberg-Parker J, Toma S (2015). Comparing the impact of course-based and apprentice-based research experiences in a life science laboratory curriculum. J Microbiol Biol Educ.

[27] Webster GF, Cummins CS (1978). Use of bacteriophage typing to distinguish Propionibacterium acne types I and II. J Clin Microbiol.

[28] Neve H, Freudenberg W, Diestel-Feddersen F, Ehlert R, Heller KJ (2003). Biology of the temperate *Streptococcus thermophilus* bacteriophage TP-J34 and physical characterization of the phage genome. Virology.

[29] Russell DA

[30] Delcher AL, Bratke KA, Powers EC, Salzberg SL (2007). Identifying bacterial genes and endosymbiont DNA with Glimmer. Bioinformatics.

[31] Besemer J, Borodovsky M (2005). GeneMark: web software for gene finding in prokaryotes, eukaryotes and viruses. Nucleic Acids Res.

[32] Lawrence JG http://cobamide2.bio.pitt.edu/computer.htm.

[33] Jordan TC, Burnett SH, Carson S (2014). A broadly implementable research course in phage discovery and genomics for first-year undergraduate students. mBio.

[34] Cresawn SG, Bogel M, Day N, Jacobs-Sera D, Hendrix RW, Hatfull GF (2011). Phamerator: a bioinformatic tool for comparative bacteriophage genomics. BMC Bioinformatics.

[35] Altschul SF, Gish W, Miller W, Myers EW, Lipman DJ (1990). Basic local alignment search tool. J Mol Biol.

[36] Söding J, Biegert A, Lupas AN (2005). The HHpred interactive server for protein homology detection and structure prediction. Nucleic Acids Res.

[37] Marchler-Bauer A, Derbyshire MK, Gonzales NR (2015). CDD: NCBI’s conserved domain database. Nucleic Acids Res.

[38] Russell DA, Hatfull GF (2017). PhagesDB: the actinobacteriophage database. Bioinformatics.

[39] Meier-Kolthoff JP, Auch AF, Klenk HP, Göker M (2013). Genome sequence-based species delimitation with confidence intervals and improved distance functions. BMC Bioinformatics.

[40] Meier-Kolthoff JP, Göker M (2017). VICTOR: genome-based phylogeny and classification of prokaryotic viruses. Bioinformatics.

[41] Lefort V, Desper R, Gascuel O (2015). FastME 2.0: a comprehensive, accurate, and fast distance-based phylogeny inference program. Mol Biol Evol.

[42] Farris JS (1972). Estimating phylogenetic trees from distance matrices. Am Nat.

[43] Letunic I, Bork P (2021). Interactive tree of life (iTOL) v5: an online tool for phylogenetic tree display and annotation. Nucleic Acids Res.

[44] Mitchell A, Chang HY, Daugherty L (2015). The InterPro protein families database: the classification resource after 15 years. Nucleic Acids Res.

[45] Bailey TJ, Elkan C (1994). Fitting a mixture model by expectation maximization to discover motifs in biopolymers. Proc Int Conf Intell Syst Mol Biol.

[46] Kumar S, Stecher G, Tamura K (2016). MEGA7: molecular evolutionary genetics analysis version 7.0 for bigger datasets. Mol Biol Evol.

[47] Källberg M, Wang H, Wang S (2012). Template-based protein structure modeling using the RaptorX web server. Nature Protoc.

[48] Jumper J, Evans R, Pritzel A (2021). Highly accurate protein structure prediction with AlphaFold. Nature.

[49] Holm L, Laiho A, Törönen P, Salgado M (2023). DALI shines a light on remote homologs: one hundred discoveries. Protein Sci.

[50] Jurrus E, Engel D, Star K (2018). Improvements to the APBS biomolecular solvation software suite. Protein Sci.

[51] Zhang Y, Skolnick J (2005). TM-align: a protein structure alignment algorithm based on the TM-score. Nucleic Acids Res.

[52] https://imagej.nih.gov/ij/.

[53] Hewetson A, Khan NH, Dominguez MJ (2020). Maturation of the functional mouse CRES amyloid from globular form. Proc Natl Acad Sci U S A.

[54] Whelly S, Johnson S, Powell J, Borchardt C, Hastert MC, Cornwall GA (2012). Nonpathological extracellular amyloid is present during normal epididymal sperm maturation. PLoS One.

[55] Wu X, Zhao Y, Sun L (2019). Crystal structure of CagV, the *Helicobacter pylori* homologue of the T4SS protein VirB8. FEBS J.

[56] Singer ZS, Ambrose PM, Danino T, Rice CM (2021). Quantitative measurements of early alphaviral replication dynamics in single cells reveals the basis for superinfection exclusion. Cell Syst.

[57] Biggs KRH, Bailes CL, Scott L, Wichman HA, Schwartz EJ (2021). Ecological approach to understanding superinfection inhibition in bacteriophage. Viruses.

[58] (2022). Carvalho C, Ren R, Han J, Qu F. Natural selection, intracellular bottlenecks of virus populations, and viral superinfection exclusion. Annu Rev Virol.

[59] Redman M, King A, Watson C, King D (2016). What is CRISPR/Cas9?. Arch Dis Child Educ Pract Ed.

[60] Cobian N, Garlet A, Hidalgo-Cantabrana C, Barrangou R (2021). Comparative genomic analyses and CRISPR-Cas characterization of *Cutibacterium acnes* provide insights into genetic diversity and typing applications. Front Microbiol.

[61] Kermani AA (2021). A guide to membrane protein X-ray crystallography. FEBS J.

[62] Marinelli LJ, Hatfull GF, Piuri M (2012). Recombineering: a powerful tool for modification of bacteriophage genomes. Bacteriophage.

[63] Knödlseder N, Nevot G, Fábrega MJ (2022). Engineering selectivity of *Cutibacterium acnes* phages by epigenetic imprinting. PLoS Pathog.

