# Supplementary material for: Putative pseudolysogeny-dependent phage gene implicated in the superinfection resistance of Cutibacterium acnes
Source: Microbiome Res Rep. 2024 Apr 18;3(3):27. doi: 10.20517/mrr.2023.42 (PMC11480721; doi:10.20517/mrr.2023.42)
Supplement: Supplementary file 1 [file mrr-3-3-27-SupplementaryMaterials.pdf]

Wottrich *et al. Microbiome Res Rep* 2024;3:27
DOI: 10.20517/mrr.2023.42

Microbiome Research Reports

Supplementary Materials

Putative pseudolysogeny-dependent phage gene implicated in the superinfection resistance of *Cutibacterium acnes*

Stephanie Wottrich[1,2], Stacee Mendonca[1], Cameron Safarpour[1], Christine Nguyen[1], Laura J. Marinelli[3], Stephen P. Hancock[4], Robert L. Modlin[3], Jordan Moberg Parker[1,5]

[1]Department of Microbiology, Immunology, and Molecular Genetics, University of California Los Angeles, Los Angeles, CA 90024, USA.

[2]Department of Neurology, Dell Seton Medical Center at the University of Texas at Austin, Austin, TX 78701, USA.

[3]UCLA Dermatology, University of California Los Angeles, Los Angeles, CA 90095, USA.

[4]Department of Chemistry, Towson University, Towson, MD 21252, USA.

[5]Department of Biomedical Science, Kaiser Permanente Bernard J. Tyson School of Medicine, Pasadena, CA 91101, USA.

Correspondence to: Dr. Stephanie Wottrich, Department of Neurology, Dell Seton Medical Center at the University of Texas at Austin, 1500 Red River St., Austin, TX 78701, USA. E-mail: stephanie.wottrich@ascension.org

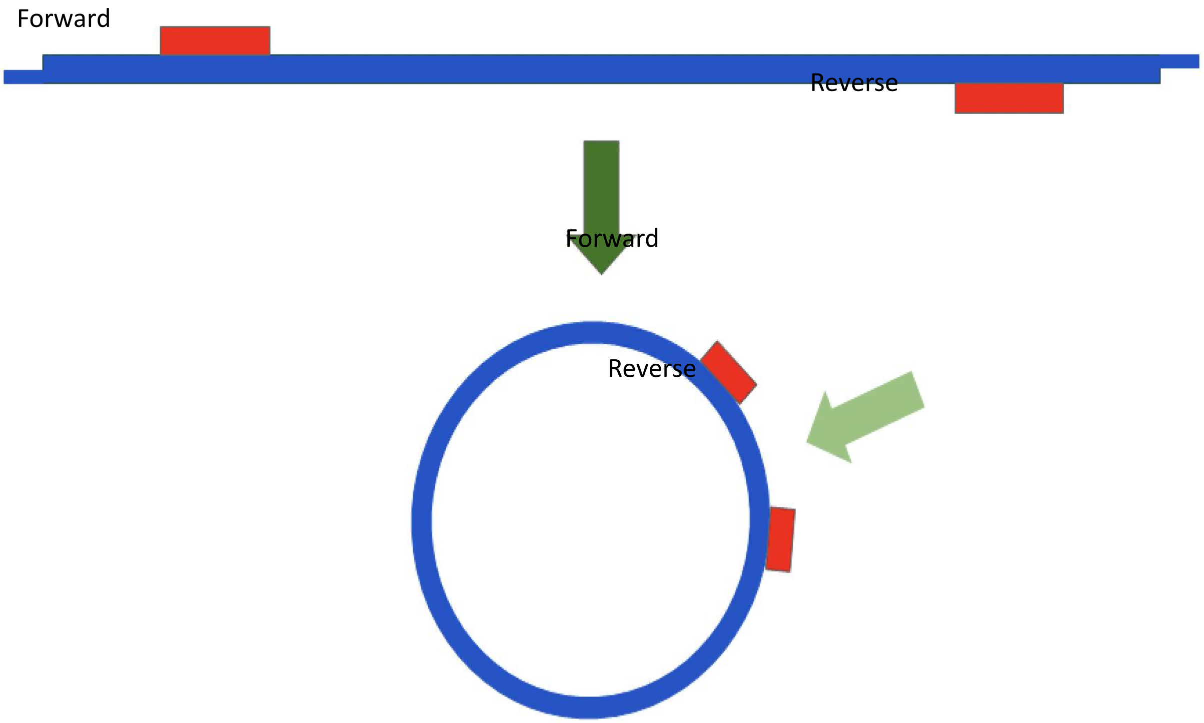

Supplementary Figure 1. Pictorial of PCR methodology supporting *C. acnes* ATCC 6919 pseudolysogeny with phage Aquarius. Shown above is an illustration of the PCR amplification process conducted on putative *C. acnes* pseudolysogens (the high stability group sample) and suspected prior pseudolysogen samples that were no longer infected by phage (the low stability group sample). The PCR sample yields a product only if the linear phage genome circularizes. This is because the unification of the 3' sticky overhang genomic ends align the 3' and 5' ends of the primers such that amplification may take place, producing a characteristically 735 bp product given the location that the primers anneal to their complementary sites (light green arrow). Primers used: Forward 5'-CCGAAGCCGACCACATCACACC-3', Reverse 5'-TCATCCAACACCTGCTGCTGCC-3'. Of note a positive control containing *M. luteus* and a negative control without phage were also analyzed, neither of which produced a 735 bp band.
